# Comparison of Clinical Features between the High and Low Serum KL-6 Patients with Acute Exacerbation of Interstitial Lung Diseases

**DOI:** 10.1155/2021/9099802

**Published:** 2021-11-30

**Authors:** Yoichi Tagami, Yu Hara, Kota Murohashi, Ryo Nagasawa, Yurika Nishikawa, Meiro Tanaka, Ayako Aoki, Katsushi Tanaka, Kentaro Nakashima, Keisuke Watanabe, Nobuyuki Horita, Nobuaki Kobayashi, Masaki Yamamoto, Makoto Kudo, Koji Okudela, Takeshi Kaneko

**Affiliations:** ^1^Department of Pulmonology, Yokohama City University Graduate School of Medicine, Yokohama, Japan; ^2^Department of Respiratory Medicine, Kanagawa Cardiovascular and Respiratory Center, Yokohama, Japan; ^3^Respiratory Disease Center, Yokohama City University Medical Center, Yokohama, Japan; ^4^Department of Pathology, Yokohama City University Graduate School of Medicine, Yokohama, Japan

## Abstract

**Background:**

Serum Krebs von den Lungen-6 (KL-6) measurement is widely used to assess disease activity or prognosis in patients with interstitial lung diseases (ILDs). However, the clinical differences between high and low serum KL-6 levels at the time of acute exacerbation (AE) of ILD are not well known.

**Methods:**

Clinical parameters including age, sex, Charlson Comorbidity Index score (CCIS), blood biomarkers, high-resolution CT findings, and disease mortality were retrospectively compared between high and low KL-6 (cutoff value: 1000 U/mL) patients at the time of diagnosis of AE of ILDs.

**Results:**

Thirty-eight high serum KL-6 and 57 low serum KL-6 patients were included. There was no significant difference in 6-month mortality between them (*P* = 0.685), whereas serum lactate dehydrogenase was a significant predictor of 6-month mortality in the high serum KL-6 patients (odds ratio (OR): 1.006; 95% confidence interval (CI): 1.003–1.009; *P* < 0.001), and CCIS (OR: 1.502; 95% CI: 1.242–1.838; *P* < 0.001) and sex (OR: 5.751; 95% CI: 1.121–105.163; *P* = 0.033) were significant predictors in low serum KL-6 patients. In addition, the incidences of congestive heart failure, symptomatic chronic pulmonary disease, cerebrovascular disease, and second metastatic solid tumours were significantly higher in nonsurvivors with low serum KL-6 than in other groups (*P* < 0.05).

**Conclusions:**

The clinical features in patients with AEs of ILDs may differ depending on the serum KL-6 level, and clinicopathological examination according to this subtyping guided by the serum KL-6 level is essential.

## 1. Introduction

The prognosis of acute exacerbations (AEs) of interstitial lung diseases (ILDs) such as idiopathic interstitial pneumonias (IIPs), chronic hypersensitivity pneumonitis, and connective tissue disease-associated ILDs (CTD-ILDs) is generally poor [1, 2]. Furthermore, the pathological findings of patients with AEs of ILDs show not only diffuse alveolar damage (DAD) but also a variety of pathological conditions including organizing pneumonia (OP), diffuse alveolar haemorrhage (DAH), lung cancer, and bronchopneumonia [3]. Despite pathological heterogeneity, it is very difficult to perform a lung biopsy during an AE due to severe respiratory failure. Therefore, it is necessary to plan the treatment strategies or attempt to accurately predict the disease prognosis using less invasive modalities such as symptoms, blood test results, and imaging findings in the clinical setting.

Krebs von den Lungen-6 (KL-6) is a high-molecular-weight mucin-like glycoprotein, also known as human mucin-1 (MUC1). It is expressed mainly on bronchiolar epithelial cells and type II pneumocytes in alveoli, particularly on proliferating and regenerating type II pneumocytes [4–6]. An official American Thoracic Society/European Respiratory Society statement proposed that a serum KL-6 level above 1000 U/mL at the initial examination in patients with stable-state ILD is associated with a worse prognosis [7–9]. In addition, a recent systematic review and meta-analysis reported that higher serum KL-6 levels were associated with an increased risk of AE of idiopathic pulmonary fibrosis (IPF) [10]. On the contrary, there are few reports of the clinical significance of the serum KL-6 level at the time of diagnosis of AEs, though we often see AEs of ILD patients with low KL-6 levels in the clinical setting [11, 12].

In the present retrospective study, clinical parameters were compared between high and low serum KL-6 patients at the time of diagnosis of AEs of ILDs to attempt to classify their clinical features according to the serum KL-6 level.

## 2. Materials and Methods

### 2.1. Study Location and Patients

The retrospective cohort study involved patients seen between 2014 and 2018 at Yokohama City University Hospital and Yokohama City University Medical Center. The medical data of 95 patients with acute or subacute IIPs, including AEs of nonspecific interstitial pneumonia and idiopathic pulmonary fibrosis (IPF), acute interstitial pneumonia, cryptogenic organizing pneumonia, drug-induced ILD, or AEs of CTD-ILDs treated with corticosteroid pulse therapy, were assessed. Patients who did not receive steroid pulse therapy or had sarcoidosis were excluded. Medical records at the time of diagnosis of AE were reviewed for data including age, sex, diagnosis of ILD, Charlson Comorbidity Index score (CCIS), blood parameters (partial pressure of oxygen in arterial blood/fraction of inspired oxygen (P/F ratio)), KL-6 (normal: <500 U/mL), lactate dehydrogenase (LDH; normal: < 225 U/L), surfactant protein-D (SP-D; normal: < 110 ng/mL), high-resolution CT (HRCT) findings, and treatment regimens, including sivelestat Na hydrate, anticoagulation therapy before steroid pulse therapy, steroid use before steroid pulse therapy, and macrolides [13]. The findings of HRCT were evaluated using the semiquantitative scoring method described by Ooi et al. [14]. Abnormalities on HRCT images of lungs were categorized as ground-glass opacity and honeycomb and scored based on the ratios (%) of the disease in each of the six lung lobes (0%: 0 points, 1–25%: 1 point, 26–50%: 2 points, 51–75%: 3 points, and 76%: 4 points). Global scores were calculated by adding the scores for each abnormality in all lobes. Patients were classified as high serum KL-6 patients (≥1000 U/mL) and low serum KL-6 patients (<1000 U/mL), and the extracted data were compared between the two groups.

### 2.2. Diagnosis of ILDs

Subtypes of IIP were confirmed from physical, serological, HRCT, and lung pathological findings in accordance with the official statement for IIPs [7, 15]. Patients for whom lung biopsy could not be performed due to severe hypoxemia were diagnosed based on the HRCT classification [7, 15]. The CTD-ILD diagnosis was confirmed by physical, serological, and HRCT findings consistent with ILD, and lung biopsy was undertaken to exclude other pulmonary diseases. A diagnosis of drug-induced ILD was based on previously reported criteria [16]. An AE of ILD was defined as worsening of hypoxemia reflecting severely impaired gas exchange; worsening of dyspnoea; newly appeared alveolar infiltration on radiography; and absence of alternative aetiologies including pneumothorax, pulmonary embolism, infection, or heart failure [7, 17–20].

### 2.3. Statistical Analysis

Data were statistically analysed using JMP 12 (SAS Institute Inc., Cary, NC, USA) and were shown as medians with 25th–75th percentiles or numbers (%). Groups were compared using the Wilcoxon rank-sum test or Pearson's chi-squared test. Optimal parameter cutoff values were determined from receiver operating characteristic (ROC) curves. Survival curves were generated using the Kaplan–Meier method and compared using log-rank tests. Predictors of 6-month mortality were determined using multiple stepwise regression analysis. Values with *P* < 0.05 were considered significant.

### 2.4. Study Approval

This research was performed in accordance with the Declaration of Helsinki and approved by the institutional review board at Yokohama City University Hospital (approval no. B171100003). In this retrospective study, consent for participation was obtained by disclosing the clinical study with the description of the opt-out process (https://www.yokohama-cu.ac.jp/amedrc /ethics/ethical/fuzoku_optout.html). The severely ill condition or deep sedation of AE-ILD patients precluded us from obtaining informed consent from the patients themselves. Therefore, written informed consent was obtained from the patients' relatives or their legal guardians.

## 3. Results

### 3.1. Patient Characteristics

**Figure 1:**
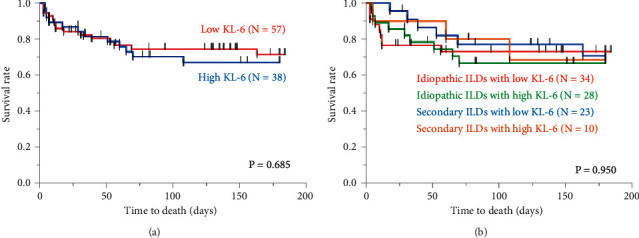
Comparison between high serum KL-6 and low serum KL-6 patients. The enrolled patients consist of 38 (40%) patients with high serum KL-6 (idiopathic: 28 patients and secondary: 10 patients) and 57 (60%) patients with low serum KL-6 (idiopathic: 34 patients and secondary 23 patients) levels. There is no significant difference in the 6-month prognosis between the high and low serum KL-6 patients (*P* = 0.685) (a). In addition, high serum KL-6 patients with AEs of idiopathic or secondary ILDs and low serum KL-6 patients with AEs of idiopathic or secondary ILDs have similar 6-month outcomes (*P* = 0.950) (b). ILD: interstitial lung disease; KL-6: Krebs von den Lungen-6.

Table 1 shows the clinical characteristics of the patients with AEs of ILDs; there were 38 (40%) patients with high serum KL-6 and 57 (60%) patients with low serum KL-6 levels. The diagnoses of the 95 patients who were all treated with corticosteroid pulse therapy were AE of idiopathic ILDs in 62 patients (65%) and AE of secondary ILDs in 33 patients (35%). There was no significant difference in the diagnoses between the high and low serum KL-6 groups. Other clinical parameters including age, sex, CCIS, symptom onset, blood biomarkers (P/F ratio and SP-D), ground-glass opacity scores calculated from HRCT, and treatment regimens except serum LDH and honeycomb score showed similar tendencies between these groups. The main cause of death in the high and low serum KL-6 groups was AE, and there was no difference in the cause of death between these two groups. High serum KL-6 patients with AEs of idiopathic or secondary ILDs and low serum KL-6 patients with AEs of idiopathic or secondary ILDs had similar 6-month mortality rates (Figure 1).

### 3.2. Stepwise Multiple Logistic Regression Analysis

**Figure 2:**
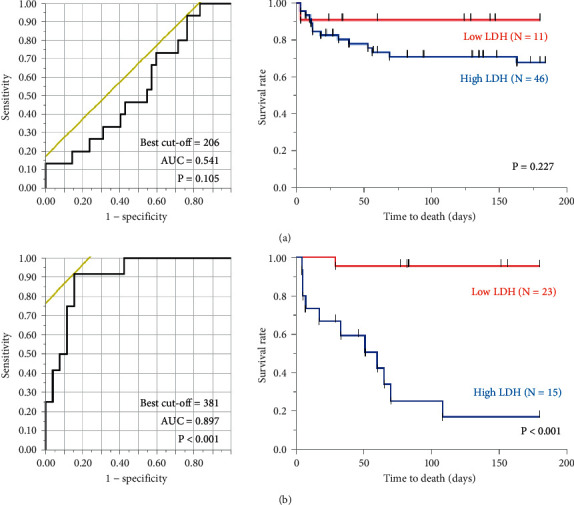
Relationship between 6-month mortality and serum LDH levels. In patients with low serum KL-6 levels, the AUC is 0.541 in the evaluation of serum LDH as a predictor of 6-month mortality (a). Log-rank tests show that the Kaplan–Meier survival curves of these groups do not differ significantly (*P* = 0.227) (A). On the contrary, in the patients with high serum KL-6 levels, the AUC value is 0.897 in the evaluation of serum LDH as a predictor of 6-month mortality (b). Log-rank tests show that the Kaplan–Meier survival curves of these groups differ significantly (*P* < 0.001) (B). AUC: area under the ROC curve; LDH: lactate dehydrogenase; KL-6: Krebs von den Lungen-6.

In both patients with high and low serum KL-6 levels, clinical parameters including age, sex, CCIS, diagnosis of ILDs, P/F ratio, serum LDH and SP-D, and the GGO and honeycomb scores were evaluated using stepwise multiple logistic regression analysis, whereas serum LDH was a significant predictor of 6-month mortality in high serum KL-6 patients (OR: 1.006; 95% CI: 1.003–1.009; *P* < 0.001), and CCIS (OR: 1.502; 95% CI: 1.242–1.838; *P* < 0.001) and sex (OR: 5.751; 95% CI: 1.121–105.163; *P* = 0.033) were significant predictors in low KL-6 patients (Table 2). In the patients with low serum KL-6 levels, the area under the ROC curve (AUC) was 0.541 in the evaluation of serum LDH as a predictor of 6-month mortality (Figure 2(a)). The 38 patients were assigned to groups with either low LDH (*N* = 11) or high LDH (*N* = 46) levels based on the optimal cutoff (206 IU/mL). Log-rank tests showed that the Kaplan–Meier survival curves of these groups did not differ significantly (*P* = 0.227) (Figure 2(a)). On the contrary, in the patients with high serum KL-6 levels, the AUC was 0.897 in the evaluation of serum LDH as a predictor of 6-month mortality (Figure 2(b)). The optimal cutoff LDH level for estimating 6-month mortality was 381 IU/mL (*P* < 0.001). The 38 patients were assigned to groups with either low serum LDH (*N* = 23) or high serum LDH (*N* = 15) levels based on this cutoff. Log-rank tests showed that the Kaplan–Meier survival curves of these groups differed significantly (*P* < 0.001) (Figure 2(b)).

### 3.3. Relationship between 6-Month Mortality and CCIS

**Figure 3:**
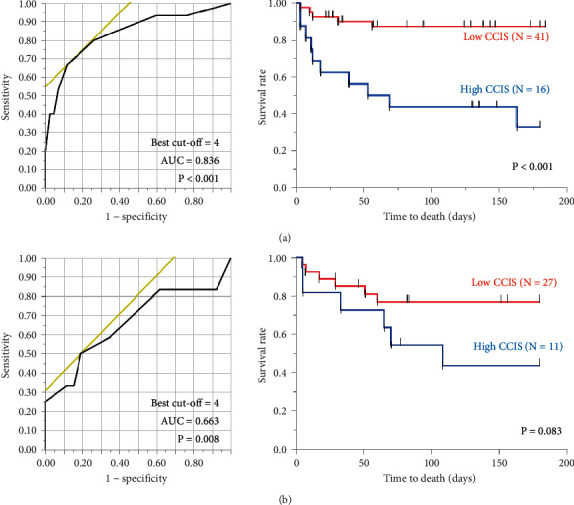
Relationship between 6-month mortality and CCIS. In the patients with low serum KL-6 levels, the AUC is 0.836 in the evaluation of CCIS as a predictor of 6-month mortality (a). The optimal cutoff CCIS for estimating 6-month mortality is 4 points (*P* < 0.001). Log-rank tests show that the Kaplan–Meier survival curves of these groups differ significantly (*P* < 0.001) (A). On the contrary, in the patients with high serum KL-6 levels, the AUC value is 0.663 in the evaluation of CCIS as a predictor of 6-month mortality (b). Log-rank tests show that the Kaplan–Meier survival curves of these groups do not differ significantly (*P* = 0.083) (B). AUC: area under the ROC curve; CCIS: Charlson Comorbidity Index score; KL-6: Krebs von den Lungen-6.

In the patients with low serum KL-6 levels, the AUC was 0.836 in the evaluation of CCIS as a predictor of 6-month mortality (Figure 3(a)). The optimal cutoff CCIS value for predicting 6-month mortality was 4 points (*P* < 0.001). The 57 patients were assigned to groups with either low CCIS (*N* = 41) or high CCIS (*N* = 16) levels based on this cutoff value. Log-rank tests showed that the Kaplan–Meier survival curves of these groups differed significantly (*P* < 0.001) (Figure 3(a)). On the contrary, in the patients with high serum KL-6 levels, the AUC was 0.663 in the evaluation of CCIS as a predictor of 6-month mortality (Figure 3(b)). The 57 patients were assigned to groups with either low CCIS (*N* = 27) or high CCIS (*N* = 11) levels based on the same cutoff value. Log-rank tests showed that the Kaplan–Meier survival curves of these groups did not differ significantly (*P* = 0.083) (Figure 3(b)).

### 3.4. Incidence of Complications according to the Serum KL-6 Level and 6-Month Outcomes

**Figure 4:**
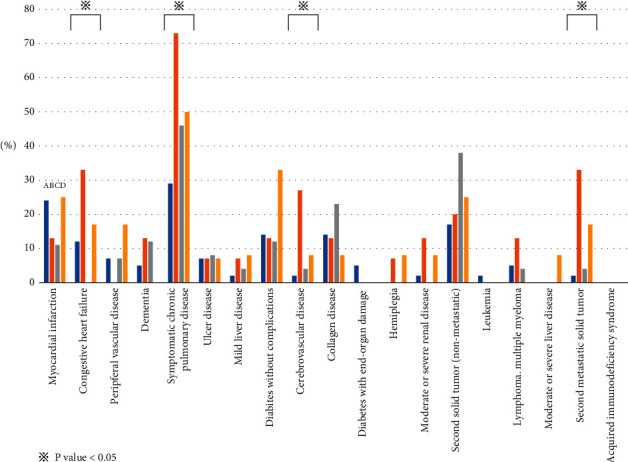
Incidence of complications according to serum KL-6 levels and 6-month outcomes. From the left bar, there are four groups, including survivors with low serum KL-6 (A), nonsurvivors with low serum KL-6 (B), survivors with high serum KL-6 (C), and nonsurvivors with high serum KL-6 (D) levels. The incidences of congestive heart failure, symptomatic chronic pulmonary disease, cerebrovascular disease, and second metastatic solid tumours are significantly the highest in nonsurvivors with low serum KL-6 levels (all *P* < 0.05). KL-6: Krebs von den Lungen-6.

Figure 4 shows a comparison of comorbidities in survivors with low serum KL-6 levels (A), nonsurvivors with low KL-6 levels (B), survivors with high serum KL-6 levels (C), and nonsurvivors with high serum KL-6 levels (D), respectively, from the left bar. The incidences of congestive heart failure (12%, 33%, 0%, and 17%), symptomatic chronic pulmonary disease (29%, 73%, 46%, and 50%), cerebrovascular disease (2%, 27%, 4%, and 8%), and second metastatic solid tumours (2%, 33%, 4%, and 17%) were the highest in nonsurvivors with low serum KL-6 levels (all *P* < 0.05).

## 4. Discussion

Serum KL-6 measurement is thought to be useful for detecting the presence of ILDs, evaluating ILD activity, and predicting the prognosis in various types of ILDs [21]. Several other clinical studies have proposed that serum KL-6 could predict the incidence of AEs, which are the most common cause of death in patients with ILD [9, 10, 22]. On the contrary, there are few reports of the relationship between the serum KL-6 levels at the time of diagnosis of AE and these disease outcomes. Though it has been reported that serum LDH (cutoff value: 280 IU/L), KL-6 (cutoff value: 1000 IU/L), P/F ratio (cutoff value: 100), and extent of abnormal HRCT findings were significant predictors of 3-month mortality in IPF patients with an AE, we often saw patients with a poor prognosis despite a normal KL-6 level at the time of AE diagnosis [11, 12]. Interestingly, in the present study, the ILD patients with high and low serum KL-6 levels had similar mortality, and it was shown that the prognostic factors were different between the two groups (high serum KL-6 group: serum LDH level; low serum KL-6 group: CCIS and sex).

A high KL-6 level was reported to be associated with the extent of lung fibrosis, which reflected regeneration of type II pneumocytes and/or enhancement of permeability following the destruction of the air-blood barrier in the affected lung [23–25]. An increased serum LDH level, which is a nonspecific biomarker, reflects lung inflammation and cellular damage in patients with ILD [26–28]. The present study showed that high serum KL-6 patients at the AE diagnosis presented a greater extent of fibrosis of HRCT, higher serum LDH levels, and a significant increase of serum KL-6 from stable condition than the low serum KL-6 patients (Supplementary Table). In addition, in the high serum KL-6 group, patients with high serum LDH levels were found to have higher GGO scores calculated from HRCT (13 points vs. 9 points (*P* < 0.001)) and lower P/F ratios (223 vs. 296 (*P* = 0.004)) than those with low serum LDH levels. From the above, patients with high serum KL-6 and LDH levels were considered to have more severe DAD with strong inflammation and increased permeability of the alveolar-capillary barrier and ongoing progressive fibrosis.

Serum KL-6 has been reported to be a significant prognostic factor in AEs of ILDs, but the serum KL-6 levels at the time of AE diagnosis are wide ranging [11]. In clinical practice, we also see patients whose serum KL-6 levels are not very high while meeting the diagnostic criteria for AE [12]. In the present study, there proved to be no difference in 6-month mortality between the high and low serum KL-6 patients. There are several possible reasons for this. First, comorbidities significantly affect the clinical course of ILD [29]. A retrospective cohort study of 272 patients with IPF suggested that there was a significant negative impact of arteriosclerosis, other cardiovascular diseases (mainly valvular heart disease, cardiac arrhythmias, and dilated cardiomyopathy), lung cancer, and pulmonary and cancer comorbidities on survival [29]. Another IPF cohort study that included 65 patients reported that baseline cardiovascular diseases were the predictors of an AE of IPF [30]. In the present study, the comparison of comorbidities between survivors with low serum KL-6, nonsurvivors with low KL-6, survivors with high serum KL-6, and nonsurvivors with high serum KL-6 levels showed that the incidences of congestive heart failure, symptomatic chronic pulmonary disease, cerebrovascular disease, and second metastatic solid tumours were significantly higher in nonsurvivors with low serum KL-6 levels than in the other groups. Second, the pathological findings in patients with AE-IPF represent not only DAD but also a variety of pathological conditions including OP, DAH, lung cancer, and bronchopneumonia [3]. In fact, comparing two autopsy cases enrolled in the present study, though HO-1, which is an oxidative stress marker, was expressed to the same extent in lung cells in both the high KL-6 case and the low KL-6 case, in the former, DAD was the main component (Supplementary Figure S1, case 1 [31]), and in the latter, DAH and pulmonary vascular microthrombosis were the main components (DAD findings were minor) (Supplementary Figure S1, case 2 [12]). Consistent with these autopsy findings, our additional data showed that hemoglobin levels with the low serum KL-6 patients were significantly lower than those with the high serum KL-6 patients (Supplementary Table). These results suggest that patients with low serum KL-6 levels do not have severe DAD and that various comorbidities and histological types such as DAH and vascular thrombosis may have a strong impact on prognosis.

The present study has some limitations. First, the study was limited by the small number of patients and the absence of additional validation datasets. In order to generalize these findings, further validation studies are essential. Second, the clinical diagnoses of the enrolled patients were heterogeneous, but there was no significant difference in the ILD diagnoses between the high and low serum KL-6 groups. Third, the low serum KL-6 group likely contained various pathological changes other than DAD, but pathological assessment was not performed after the onset of AE in all patients due to severe respiratory failure. Therefore, the credibility of this study will be increased by evaluating the relationship between clinical parameters such as blood examination and radiographic findings and prognosis in autopsy cases only.

## 5. Conclusions

The clinical features of patients with AEs of ILDs may differ depending on the serum KL-6 level, and clinicopathological examination according to this subtyping guided by the serum KL-6 level is essential.

## Figures and Tables

**Table 1:** Patients' characteristics.

Characteristics	High serum KL-6 (*N* = 38)	Low serum KL-6 (*N* = 57)	Total patients (*N* = 95)	*P* values (high KL-6 vs. low KL-6)
Age, y	75 (71–80)	76 (70–81)	75 (71–80)	0.761
Male sex	24 (63)	44 (77)	68 (72)	0.137
CCIS	2 (1–4)	2 (1–4)	2 (1–4)	0.832
From symptom onset to treatment, days	7 (2.5–19)	6 (2.5–14)	6 (2.8–15)	0.840

Diagnosis of AE				
Idiopathic ILDs				
IPF	8 (21)	9 (16)	17 (18)	0.589
Others	20 (53)	25 (44)	45 (47)	0.412
Secondary ILDs				
CTD-ILD	6 (16)	13 (23)	19 (20)	0.445
Drug	3 (8)	10 (17)	13 (14)	0.232
Others	1 (2)	0 (0)	1 (1)	0.400

Biomarkers				
P/F ratio	274 (218–308)	248 (152–311)	268 (187–309)	0.329
LDH, IU/L	321 (268–446)	277 (216–375)	282 (235–405)	0.013
SP-D, ng/mL	337 (137–541)	207 (140–364)	233 (138–409)	0.112

*HRCT scores*				
GGO scores	10 (6–13.8)	10 (8–15.5)	10 (7–15)	0.729
Honeycomb	3 (0–7)	0 (0–4)	1 (0–5)	0.045

Treatment				
PSL before pulse	11 (29)	10 (18)	21 (22)	0.189
PSL pulse	38 (100)	57 (100)	95 (100)	1.000
PSL after pulse	11 (29)	23 (40)	34 (36)	0.256
Macrolide	8 (21)	12 (21)	20 (21)	1.000
NEI	3 (8)	8 (14)	11 (12)	0.360
Anticoagulant	6 (16)	12 (21)	18 (19)	0.521

Outcome				
Six-month mortality	12 (32)	15 (26)	27 (28)	0.685

Cause of mortality				
AE	11 (29)	14 (25)	25 (26)	0.634
Lung cancer	1 (3)	1 (2)	2 (2)	0.771

Results are shown as medians with 25th–75th percentiles or numbers (%). Serum SP-D could be measured in 92 patients (97%). AE: acute exacerbation; CCIS: Charlson Comorbidity Index score; CVD-IP: collagen vascular disease-related interstitial pneumonia; GGO: ground-glass opacity; HRCT: high-resolution computed tomography; ILD: interstitial lung disease; IPF: idiopathic pulmonary fibrosis; KL-6: Krebs von den Lungen-6; LDH: lactate dehydrogenase; NEI: neutrophil elastase inhibitor; P/F ratio: partial pressure of oxygen in arterial blood/fraction of the inspiratory oxygen; PSL: prednisolone; SP-D: surfactant protein-D.

**Table 2:** Multiple stepwise regression analysis of primary predictors of 6-month mortality (age, sex, CCIS, diagnosis, LDH, P/F ratio, ground-glass opacity, and honeycomb scores).

Variable	95% confidence interval	Odds ratio	*P* values
*(A) Low KL-6 group*			
CCIS	1.502	1.242–1.838	<0.001
Sex, male vs. female	5.751	1.121–105.163	0.033
Serum LDH	1.002	1.000–1.005	0.058

*(B) High KL-6 group*			
Serum LDH	1.006	1.003–1.009	<0.001

CCIS: Charlson Comorbidity Index score; LDH: lactate dehydrogenase; P/F ratio: partial pressure of oxygen in arterial blood/fraction of inspired oxygen.

## Data Availability

The datasets used and/or analysed during the current study are available from the corresponding author upon reasonable request.

## Abbreviations

AE: Acute exacerbation
AUC: Area under the ROC curve
CCIS: Charlson Comorbidity Index score
CI: Confidence interval
CTD-ILD: Connective tissue disease-associated ILD
DAD: Diffuse alveolar damage
DAH: Diffuse alveolar haemorrhage
GGO: Ground-glass opacity
HRCT: High-resolution CT
HO-1: Heme oxygenase-1
IIPs: Idiopathic interstitial pneumonias
ILD: Interstitial lung disease
IPF: Idiopathic pulmonary fibrosis
KL-6: Krebs von den Lungen-6
LDH: Lactate dehydrogenase
NEI: Neutrophil elastase inhibitor
OP: Organizing pneumonia
OR: Odds ratio
P/F ratio: Partial pressure of oxygen in arterial blood/fraction of inspired oxygen
PSL: Prednisolone
ROC: Receiver operating characteristic
SP-D: Surfactant protein-D.

## Supplementary Materials

Supplementary Materials Pathological findings at autopsy of AEs of ILDs [12, 31]. Figure legends: case 1 is a patient with high serum KL-6 (A: HE staining; C: immunohistochemical staining for HO-1 expression) [31]. Case 2 is a patient with low serum KL-6 (B: HE staining; D: immunohistochemical staining for HO-1 expression) [12]. (A) In addition to the fibrotic lesions that had passed for a relatively long time, oedematous thickening of the alveolar septum and vitreous membrane formation are observed focally. There is no evidence of granulomas or vasculitis. This is considered consistent with active DAD superimposed on fibrosis. (B) DAH and relatively mild DAD-like change are superimposed on fibrosis. DAH is the more predominant pattern than DAD. In addition, pulmonary vascular thrombosis is evident. This case is considered to be not typical for AE of ILD. (C, D) In both cases, high expression of HO-1 is similarly observed mainly in alveolar macrophages, whereas the expression of HO-1 in fibrotic lesions is not conspicuous. Abbreviations: AE: acute exacerbation; DAD: diffuse alveolar damage; DAH: diffuse alveolar haemorrhage; HE: haematoxylin-eosin staining; HO-1: heme oxygenase-1; KL-6: Krebs von den Lungen-6; ILD: interstitial lung disease. Supplementary Table: footnotes: ΔKL-6 means the variation of serum KL-6 from a stable condition to acute exacerbation diagnosis. These data could be assessed in 52 patients (high KL-6 patients: 25 and low KL-6 patients: 27). D-dimer level could be measured in 78 patients (high KL-6 patients: 28 and low KL-6 patients: 50). Abbreviations: Hb: hemoglobin.
