# Supplementary material for: Comparison of Clinical Features between the High and Low Serum KL-6 Patients with Acute Exacerbation of Interstitial Lung Diseases
Source: Can Respir J. 2021 Nov 30;2021:9099802. doi: 10.1155/2021/9099802 (PMC8651422; doi:10.1155/2021/9099802)
Supplement: Supplementary Materials — Pathological findings at autopsy of AEs of ILDs [12, 31]. Figure legends: case 1 is a patient with high serum KL-6 (A: HE staining; C: immunohistochemical staining for HO-1 expression) [31]. Case 2 is a patient with low serum KL-6 (B: HE staining; D: immunohistochemical staining for HO-1 expression) [12]. (A) In addition to the fibrotic lesions that had passed for a relatively long time, oedematous thickening of the alveolar septum and vitreous membrane formation are observed focally. There is no evidence of granulomas or vasculitis. This is considered consistent with active DAD superimposed on fibrosis. (B) DAH and relatively mild DAD-like change are superimposed on fibrosis. DAH is the more predominant pattern than DAD. In addition, pulmonary vascular thrombosis is evident. This case is considered to be not typical for AE of ILD. (C, D) In both cases, high expression of HO-1 is similarly observed mainly in alveolar macrophages, whereas the expression of HO-1 in fibrotic lesions is not conspicuous. Abbreviations: AE: acute exacerbation; DAD: diffuse alveolar damage; DAH: diffuse alveolar haemorrhage; HE: haematoxylin-eosin staining; HO-1: heme oxygenase-1; KL-6: Krebs von den Lungen-6; ILD: interstitial lung disease. Supplementary Table: footnotes: ΔKL-6 means the variation of serum KL-6 from a stable condition to acute exacerbation diagnosis. These data could be assessed in 52 patients (high KL-6 patients: 25 and low KL-6 patients: 27). D-dimer level could be measured in 78 patients (high KL-6 patients: 28 and low KL-6 patients: 50). Abbreviations: Hb: hemoglobin. [file 9099802.f1.zip › 9099802.f1/Supplement figure 20211118.docx]

**Supplementary figure**

**Pathological findings at autopsy of AEs of ILDs [12, 31]**

**
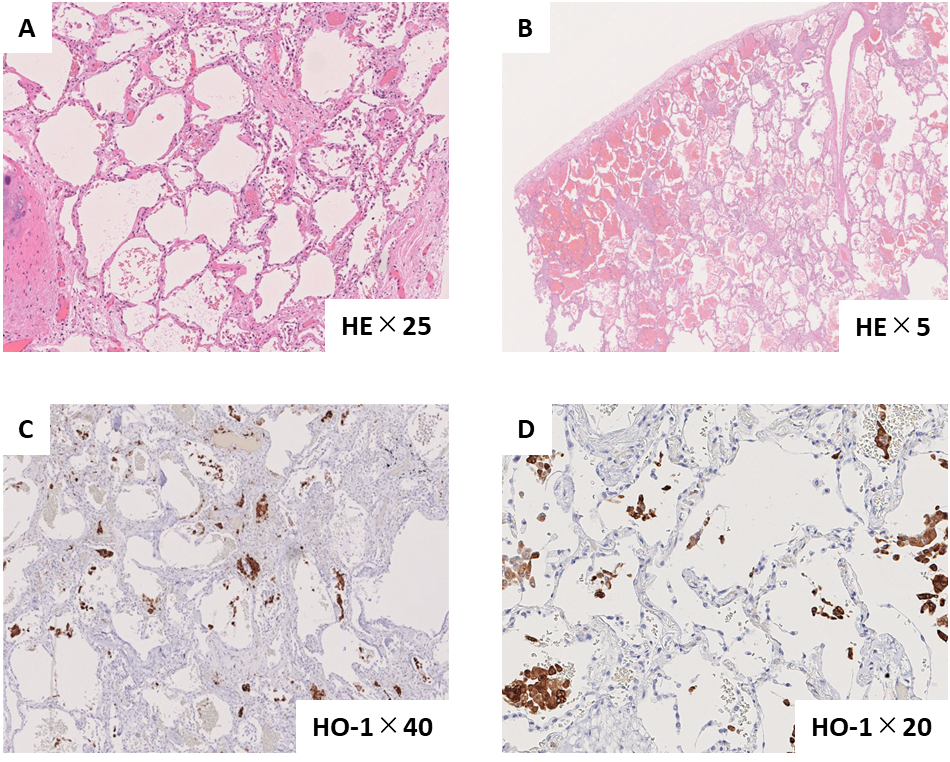
** **Figure legends**

Case 1 is a patient with a high serum KL-6 (A: HE staining, C: Immunohistochemical staining for HO-1 expression) [31].

Case 2 is a patient with a low serum KL-6 (B: HE staining, D: Immunohistochemical staining for HO-1 expression) [12].

(A) In addition to the fibrotic lesions that had passed for a relatively long time, oedematous thickening of the alveolar septum and vitreous membrane formation are observed focally. There is no evidence of granulomas or vasculitis. This is considered consistent with active DAD superimposed on fibrosis.

(B) DAH and relatively mild DAD-like change are superimposed on fibrosis. DAH is the more predominant pattern than DAD. In addition, pulmonary vascular thrombosis is evident. This case is considered to be not typical for AE of ILD.

(C, D) In both cases, high expression of HO-1 is similarly observed mainly in alveolar macrophages, whereas expression of HO-1 in fibrotic lesions is not conspicuous.

Abbreviations: AE, acute exacerbation; DAD, diffuse alveolar damage; DAH, diffuse alveolar haemorrhage; HE, haematoxylin-eosin staining; HO-1, hemeoxygenase-1; KL-6, Krebs von den Lungen; ILD, interstitial lung disease.
